# 朗格汉斯细胞组织细胞增生症的免疫微环境：从免疫抑制到靶向治疗

**DOI:** 10.3760/cma.j.cn121090-20241030-00425

**Published:** 2025-07

**Authors:** 铮铮 刘, 欣欣 曹

**Affiliations:** 1 国家癌症中心、国家肿瘤临床医学研究中心、中国医学科学院北京协和医学院肿瘤医院内科，北京 100021 Department of Medical Oncology, National Cancer Center/National Clinical Research Center for Cancer/Cancer Hospital, Chinese Academy of Medical Sciences & Peking Union Medical College, Beijing 100021, China; 2 中国医学科学院、北京协和医学院北京协和医院血液内科，北京 100730 Department of Hematology, Peking Union Medical College Hospital, Chinese Academy of Medical Sciences & Peking Union Medical College, Beijing 100730, China

## Abstract

朗格汉斯细胞组织细胞增生症（Langerhans cell histiocytosis，LCH）是一种罕见的血液系统疾病，其特征是肿瘤性树突状细胞（DC）的克隆性增生，这些DC表现出未成熟和衰老的免疫表型，而背景中具有抑制功能的调节性T（Treg）细胞增加，呈耗竭状态的CD8^+^ T细胞和髓源性抑制细胞进一步加剧了免疫抑制，构成LCH独特的抑制性免疫微环境，从而有助于肿瘤DC的免疫逃逸。当前LCH治疗面临复发和耐药挑战，而靶向肿瘤DC衰老表型和Treg细胞、逆转T细胞耗竭等免疫检查点阻断策略为LCH治疗提供了新方向。

## Abstract (en)

Langerhans cell histiocytosis（LCH）is a rare hematologic disorder characterized by the clonal proliferation of neoplastic dendritic cells（DCs）, exhibiting both immature and senescent immune phenotypes. The immunosuppressive microenvironment in LCH includes an increased proportion of regulatory T（Treg）cells with inhibitory functions, as well as exhausted CD8^+^ T cells and myeloid-derived suppressor cells, which collectively exacerbate immunosuppression and facilitate the immune evasion of tumor DCs. Current therapeutic approaches for LCH are limited by the challenges of relapse and drug resistance. However, emerging strategies that target the senescent phenotype of neoplastic DCs, inhibit Treg cell activity, and reverse T cell exhaustion through immune checkpoint blockade offer promising avenues for the treatment of LCH.

朗格汉斯细胞组织细胞增生症（Langerhans cell histiocytosis，LCH）是一种源于树突状细胞（dendritic cell，DC）的血液系统疾病，尽管发病率低、临床罕见，但研究人员从未停止对其疾病本质、发病机制及治疗方案的探究，从最初的“组织细胞增生症X”到目前公认的“炎性髓系肿瘤”，认识逐步深入。一方面伴有重现性丝裂原活化蛋白激酶（mitogen-activated protein kinase，MAPK）通路突变的CD207^+^ DC决定了其肿瘤性质[1]，另一方面“炎性”二字强调LCH中大量的免疫细胞如T细胞、B细胞、浆细胞、粒细胞、髓源性抑制细胞（myeloid-derived suppressor cell，MDSC）等构成的微环境，其细胞组成和功能特征可能与其他类型肿瘤的微环境不同。目前关于LCH研究的焦点多集中于肿瘤DC分离和鉴定、活化的分子通路以及治疗方案的优化等方面，而微环境相关研究甚少。其是“旁观者”还是“参与幕后决策”？肿瘤DC发生逃逸时各种细胞的具体作用以及肿瘤DC与微环境的调控关系等问题逐渐成为研究人员的研究方向。

现有研究显示LCH中的DC是功能异常的抗原呈递细胞（antigen presenting cell，APC），呈现衰老表型，微环境中的细胞特别是T细胞也数量失衡、功能失调并呈现为耗竭表型[2]，而近期研究显示程序性死亡受体1（programmed death-1，PD-1）抗体可在一定程度逆转LCH中的耗竭表型T细胞，恢复其功能，从而清除肿瘤DC[3]。本文就LCH中肿瘤DC的衰老表型、与微环境的调控关系、T细胞耗竭表型相关研究及进展作如下综述。

一、LCH中的肿瘤DC

LCH与正常皮肤朗格汉斯细胞（Langerhans cell，LC）有共同的标志物（S100、CD1a和CD207），但形态上肿瘤DC缺少成熟DC的特征性突起，因此研究多集中于比较细胞表面共刺激分子CD40/CD80/CD86和MHC Ⅰ、Ⅱ类等抗原表达及功能，并提出不成熟DC活化模型（Activated-Immature Model）[4]，而肿瘤DC的不成熟表型可能与病变部位[5]–[7]及LCH突变状态有关[8]。

Allen等[9]用流式细胞术结合基因芯片技术首次建立了LCH患者病变组织中CD207^+^细胞的基因表达图谱，发现和健康人皮肤LC的表型区别并非为上述共刺激分子，而是过表达早期不成熟髓系抗原。这为LCH的“异常导向的骨髓DC前体（Misguided Myeloid Dendritic Cell Precursor）模型”提供了证据。更重要的是，这些细胞高表达分泌型磷蛋白1（secreted phosphoprotein 1，SPP1），其具有与CD44复合物结合抑制T细胞的活化[10]、调节CD8^+^ T细胞的功能[11]，从而促进肿瘤的免疫逃逸。衰老相关基因细胞周期蛋白依赖性激酶抑制因子2A（cyclin-dependent kinase inhibitor 2A，CDKN2A）以及基质金属蛋白酶（matrix metalloproteinase，MMP）9表达亦高于正常对照[9]–[10]。2022年单细胞测序[12]结果显示，LCH中MHC Ⅰ类抗原表达水平低，但PD-L1和PD-L2及一系列衰老相关基因及蛋白，如CDKN2A、核层蛋白A、P21激活激酶、P16、哺乳动物雷帕霉素靶蛋白（mammalian target of rapamycin，mTOR）和P53等表达水平高。上述的衰老相关基因及蛋白驱动了衰老相关分泌表型（senescence-associated secretory phenotype，SASP）在LCH病变中聚集。

总之，目前对LCH肿瘤DC的认知除不成熟表型外，更多地认为其是一群表型和功能异常的衰老型APC。

二、LCH微环境的特殊性

除了肿瘤DC外，LCH病变中还存在多种细胞，占50％～90％，其中占比最多的是T细胞。生理状态下，成熟DC表面高表达CD40、CD80（B7-1）、CD86（B7-2）等，可与T细胞表面的正性刺激分子CD40L、CD28以及CTLA-4（即CD152）形成CD40-CD40L、CD28-CD80/CD86或CD152-CD80/CD86受体-配体复合物，其相互作用可调节T细胞功能，CD40-CD40L可双向激活APC和T细胞。LCH中的肿瘤DC存在表型及功能障碍，其无法正常执行抗原呈递作用，从而导致肿瘤微环境的混乱[6]–[7],[13]–[14]。

（一）调节性T（Treg）细胞

Treg细胞是具有免疫抑制功能的T细胞亚群，其特异性表型为CD4^+^CD25^+^叉头框蛋白3（forkhead box protein 3，FOXP3）^+^。Allen等[9]对比LCH外周血及病变组织CD3^+^细胞的基因表达谱，发现病变组织CD3^+^细胞表面FOXP3、CTLA-4的表达以及SPP1的分泌均有所增加，提示Treg细胞被激活，免疫抑制功能增强。另有研究结果显示LCH患者中Treg细胞明显扩增，可占T细胞的20％[15]。肿瘤DC可以通过表达NF-κB受体激活蛋白来诱导LCH病变内Treg细胞的积累，而Treg细胞的增多增强了机体的免疫抑制功能，使其无法对肿瘤DC产生有效的免疫应答，而免疫逃逸的肿瘤DC可继续增加Treg细胞的数量，进而形成恶性循环[15]。另外，尽管磷脂酰肌醇3激酶（phosphoinositide 3-kinase，PI3K）通路突变在LCH肿瘤DC中罕见[16]，但微环境中Treg细胞的发育与分化的调节高度依赖PI3K信号转导[17]。以上表明，Treg细胞可能通过其免疫抑制功能在LCH发生发展中起作用[18]。

（二）CD8^+^细胞毒性T细胞（CTL）

许多恶性肿瘤中，浸润的T细胞可介导适应性免疫反应并清除肿瘤细胞，CTL为主要的效应细胞。尽管最近研究显示CD4^+^ CTL也具有很强的抗肿瘤能力[19]，但多数CTL是由CD8^+^ T细胞分化而来。LCH病变中活化的CTL持续表达抑制性受体，包括CTLA-4、PD-1、细胞毒性T细胞免疫球蛋白黏蛋白分子3（T-cell immunoglobulin and mucin domain-containing molecules 3，TIM-3）、淋巴细胞活化基因3（lymphocyte activation gene-3，LAG-3）和T细胞免疫球蛋白ITIM结构域[20]–[21]，导致CTL效应功能下降，增殖能力进行性丢失，并伴随转录组和表观遗传学的改变，从而出现T细胞耗竭。

LCH中也可见CD8^+^ T细胞浸润，其效应发挥需要与MHC Ⅰ类分子结合，但由于肿瘤DC表面共刺激分子和MHC Ⅰ类抗原表达水平低，CD8^+^细胞活化受限，因此与上述功能正常的Treg细胞群不同的是：LCH中大多数CD8^+^ CTL细胞存在功能障碍。Sengal等[3]的研究结果显示LCH病变中CD8^+^ CTL细胞功能低下，而且即使加入外源性刺激，其γ干扰素（IFN-γ）、IL-17、TNF-α以及IL-10的表达水平依然很低，与之对应的异体反应性不佳。在LCH病变中CD8^+^细胞表面抑制性受体的表达显著增加，特别是PD-1、TIM-3和LAG-3的表达升高。抑制性受体的上调导致T细胞活化阈值的提高，继而导致抑制性免疫反应及T细胞耗竭。进一步评估显示，CD8^+^细胞抑制性受体增加，且其CTL杀伤功能性标志物即颗粒酶B和穿孔素的分泌明显降低，提示CD8^+^ CTL为耗竭表型[3]。此外，肿瘤DC表达高水平PD-L1和PD-L2，与CD8^+^细胞表面PD-1相结合，进一步抑制了CD8^+^细胞的免疫功能。这种受抑制的免疫反应促进了T细胞耗竭，形成利于肿瘤DC免疫逃逸的微环境[3]。

（三）其他细胞

除T细胞外，LCH背景中还伴有较多的嗜酸性粒细胞和中性粒细胞，而中性粒细胞作为髓系细胞的主要成分，在免疫调控过程中扮演着重要角色。病理性激活的中性粒细胞称为多形核MDSC，具有强大的免疫抑制活性[22]，其发挥抑制作用一方面是自身分泌抑制性因子，另一方面通过转化生长因子β（TGF-β）依赖方式或分泌的IL-10/IFN-γ诱导Treg细胞扩增，MDSC和Treg细胞抑制作用叠加，从而形成更为强大的免疫耐受微环境。

（四）细胞因子

另外，LCH病变中肿瘤DC及微环境中的细胞可分泌大量细胞因子，其中免疫抑制性细胞因子可能进一步加剧T细胞的耗竭状态形成。

1. IL-17A：IL-17A主要由Th17细胞产生，某些情况下巨噬细胞、中性粒细胞和嗜酸性粒细胞等髓样细胞也可产生，最初研究显示活动期LCH患者血清中存在高水平IL-17A且其主要来源于肿瘤DC[23]。但后续RNA及蛋白质水平的研究与质谱分析[24]–[25]均未在肿瘤DC上检测出IL-17A的存在。尽管IL-17A的确切来源仍有争议，但其在活动期LCH患者血清中高水平表达已得到广泛认可。目前认为IL-17A可促进DC融合产生小的多核巨噬细胞，进而表达酸性磷酸酶及与SASP相关的MMP9和MMP12[23]。以上提示IL-17A可能在维持肿瘤DC的衰老表型中担任重要角色。

2. IL-10：活动期LCH中，巨噬细胞、T细胞[15],[26]与嗜酸性粒细胞[27]可分泌高水平IL-10，进而下调B7分子和MHC Ⅱ类分子的表达使肿瘤DC停滞在未成熟阶段。在孤立或愈合的皮肤病变中IL-10表达缺失，肿瘤DC则表现出更成熟的表型，提示IL-10下调可促进其成熟[7],[28]。LCH病变中IL-10的表达还与Treg细胞的CD25和诱导性共刺激分子表达升高有关，可进一步维持LCH抑制性微环境[6]–[7]。此外，临床病例报道和研究显示[29]–[30]，LCH患者外周血中高水平IL-10一定程度上提示患者对化疗反应差，并与疾病进展呈正相关。

3. TGF-β1：与IL-10功能相似，TGF-β1同样可以通过抑制MHC Ⅱ类分子、CD80、CD86和CD83的表达，阻碍DC成熟。其可以促进单核细胞向LC分化，并通过抑制非特异性信号（如脂多糖、TNF-α和IL-1等）引发的成熟过程来维持这些细胞的未成熟状态，有助于肿瘤DC在病变部位的积累[31]–[32]。另外，TGF-β1可以增强LC的内吞作用，使其维持较高的活性，这也是未成熟DC的特征[32]。

4. TNF-α：TNF-α不仅直接或间接促进DC细胞成熟[6]，还参与了炎症反应和细胞凋亡的调控，是关键的SASP相关细胞因子。尽管在微环境中TNF-α的水平可能较高，但肿瘤DC依然维持不成熟表型[33]，在这种情况下，TNF-α产生了加重炎症反应的“副作用”。

5. 其他：LCH的免疫微环境中还存在其他多种细胞因子，如IL-1β能够帮助诱导B7分子表达，有助于LC的成熟和存活，与TNF-α、TGF-β1共同调控LC的成熟状态和功能[6]。粒细胞-巨噬细胞集落刺激因子（GM-CSF）和IL-1β可以协同促进LC表面B7分子表达，进而促进LC迁移和成熟[6]。GM-CSF和TNF-α可在体外共同诱导造血干祖细胞分化产生LC，并且其在体内也能激活LC，导致LC局部聚集[32],[34]。在患者体内，LC的密度与局部GM-CSF的存在密切相关，此外，在活动性和播散性LCH患儿的血清中可以检测到GM-CSF的存在，表明其水平与疾病的范围和活动性有关[34]。

综上所述，LCH微环境中，具有抑制功能的Treg细胞占主导地位，CD8^+^ CTL虽具强效抗肿瘤潜能，但LCH中的CTL呈耗竭表型、功能缺陷，以上同MDSC及多种抑制性细胞因子构成了LCH独特的抑制性免疫微环境（图1）。

**图1:**
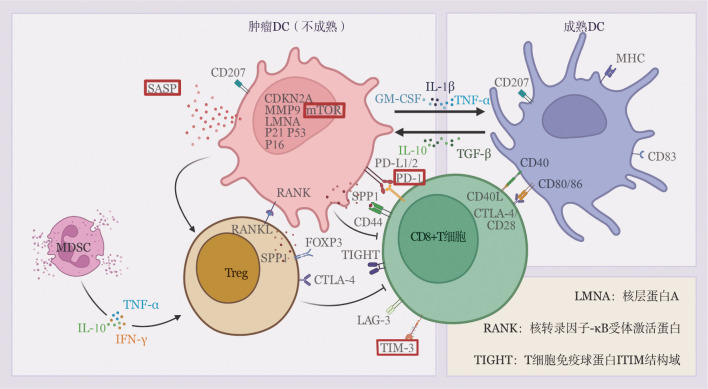
朗格汉斯细胞组织细胞增生症免疫微环境中肿瘤树突状细胞的表型差异及其与免疫细胞的相互作用 **注** DC：树突状细胞；Treg：调节性T细胞；MDSC：髓源性抑制细胞；GM-CSF：粒细胞-巨噬细胞集落刺激因子；TGF-β：转化生长因子β；TNF-α：肿瘤坏死因子-α；IFN-γ：γ干扰素；SASP：衰老相关分泌表型；CDKN2A：细胞周期蛋白依赖性激酶抑制剂2A；MMP9：基质金属蛋白酶9；mTOR：哺乳动物雷帕霉素靶蛋白；SPP1：分泌型磷蛋白1；FOXP3：叉头框蛋白3；PD-1：程序性死亡受体1；LAG-3：淋巴细胞活化基因3；TIM-3：细胞毒性T细胞免疫球蛋白黏蛋白分子3；CTLA-4：细胞毒性T淋巴细胞相关抗原4；MHC：主要组织相容性复合体

三、靶向衰老DC和免疫微环境是具有前景的LCH治疗新方向

目前有多种MAPK通路抑制剂可用于伴有MAPK通路异常的LCH患者[35]，包括BRAF抑制剂维莫非尼[36]–[37]和达拉非尼[38]–[41]、丝裂原活化蛋白激酶/细胞外信号调节激酶激酶（Mitogen-activated protein kinase/Extracellular signal-regulated kinase Kinase，MEK）抑制剂曲美替尼[38]–[39]，它们的疗效都已在临床试验中被证实。然而，治疗中仍存在无MAPK通路异常患者的限制使用以及患者停药后复发及耐药等问题。

（一）靶向衰老肿瘤DC可能是有效的治疗选择

LCH中肿瘤DC呈衰老表型，衰老细胞中mTOR通路持续激活并产生大量SASP，进而影响造血祖细胞向单核吞噬细胞（mononuclear phagocyte，MNP）的分化[2]。因此，Bigenwald团队应用mTOR抑制剂雷帕霉素治疗BRAF V600E Scl^+^小鼠，发现其可以通过抑制肿瘤DC分泌SASP，减少骨髓中粒细胞-巨噬细胞祖细胞和MNP的积累，减轻器官肿大、减少组织中的炎症细胞浸润，进而使LCH症状得到缓解[2]。尽管缺乏使用雷帕霉素治疗LCH的临床数据，但已有其治疗Erdheim-Chester病的前瞻性研究[42]，其也可用于治疗幼年型黄色肉芽肿[43]、良性头部组织细胞增生症[43]–[44]和Rosai-Dorfman病[45]。

TNF-α作为关键的SASP相关细胞因子，TNF-α抑制剂（如英夫利西单抗、依那西普）在临床前模型中显示出一定疗效[46]，但LCH患者的治疗效果存在差异[47]–[48]，需进一步试验，判断其临床应用价值。

（二）靶向Treg细胞及逆转T细胞耗竭可提高LCH的疗效

随着免疫检查点阻断疗法在各种恶性肿瘤中广泛应用，T细胞耗竭特别是CD8^+^ T细胞耗竭逐渐引起关注[49]。LCH微环境中的CD8^+^ T呈现独特的耗竭表型，而PD-1抗体可在一定程度上修复其效应功能。Sengal等[3]在LCH动物模型中的研究结果显示，MEK抑制剂可减少病变中的髓系成分，但却导致CD8^+^ T细胞数量增加，而这些耗竭型T细胞功能受限，无法对肿瘤细胞产生反应；联合抗PD-1治疗后，T细胞功能恢复，提示靶向微环境的治疗可与MAPK抑制剂产生协同作用，提高LCH患者的疗效[3]。在临床应用中，PD-1抑制剂在LCH中的效果还在探索阶段。有报道显示，播散性黄色肉芽肿患者使用PD-1抑制剂后虽初期缓解，但因心脏不良反应终止治疗，提示免疫治疗的耐受性问题[50]。然而，也有组织细胞肉瘤（histiocytic sarcoma，HS）患者接受nivolumab治疗后病情得到改善或接受PD-1抑制剂联合放疗后病情稳定的个例报道[51]–[52]，表明免疫疗法在组织细胞疾病中可能是有效且耐受性好的治疗选择。

信号调节蛋白α（signal regulatory protein α，SIRPα）主要表达于树突细胞、巨噬细胞、中性粒细胞等髓系细胞，与CD47结合可调节DC的成熟和迁移，从而影响T细胞的激活和Treg细胞的功能[53]。Okamoto等[53]证实了抗SIRPα的单克隆抗体在LCH小鼠模型中的治疗效果，从而为LCH的治疗提供了一种新的策略。另有研究显示活动性LCH患者的Treg细胞比例与血浆中TIM-3水平显著相关[54]。TIM-3也可作为新的免疫检查点[55]。

除此之外，PI3Kδ作为T细胞信号传导的重要组成部分，其激活状态与T细胞耗竭的机制密切相关[56]，PI3Kδ过度激活可导致CD8^+^ T细胞的耗竭[57]–[59]。使用PI3Kδ抑制剂能够减少Treg细胞数量、增强CD8^+^ T细胞功能，从而改变肿瘤微环境以提高机体的抗肿瘤能力[60]。以上针对特异性靶点的抑制剂虽尚未用于LCH治疗，但鉴于T细胞耗竭与LCH的密切关系，可以预见其在LCH中的应用前景。

四、总结

本综述重点分析了LCH中肿瘤DC的异常特征、与免疫微环境中细胞成分及分泌因子间的相互作用，随着相关研究的逐步深入，对LCH的治疗理念将进一步拓宽。以MAPK抑制剂为主要治疗方案的基础上，可尝试增加靶向LCH肿瘤DC衰老表型、Treg细胞和逆转T细胞耗竭的药物。
